# Clinicopathological Prognostic Factors Influencing Survival Outcomes of Vulvar Cancer

**DOI:** 10.31557/APJCP.2021.22.8.2541

**Published:** 2021-08

**Authors:** Monwanee Muangchang, Prapaporn Suprasert, Surapan Khunamornpong

**Affiliations:** 1 *Division of Gynecologic Oncology, Department of Obstetrics and Gynecology, Faculty of Medicine, Chiang Mai University, Chiang Mai, Thailand. *; 2 *Division of Gynecologic Pathology, Department of Pathology, Faculty of Medicine, Chiang Mai University, Chiang Mai, Thailand. *

**Keywords:** Clinico, pathological factors, survival outcome, vulva cancer

## Abstract

**Background:**

The prognostic factors for survival in squamous cell carcinoma (SCCA) of vulva cancer such as groin node involvement, postmenopausal status, tumor size, margin status, tumor grade, lymph vascular space invasion (LVSI) were reported in the past. However, with limited data from Southeast - Asian population, the present study was conducted to evaluate the clinicopathological prognostic factors for survival outcomes of this disease after treatment with surgery.

**Methods:**

All SCCA vulva cancer patients who underwent surgery between January 2006 and December 2017 were reviewed. The clinicopathological factors were analyzed to identify the prognostic factors for the progression-free survival (PFS) and overall survival (OS) using the Kaplan- Meier method and Cox-Proportional Hazard model.

**Results:**

One hundred twenty-five patients were recruited. The independent poor prognostic factors for PFS were groin node-positive and pathologic tumor diameter of more than 25 mm. Whereas postmenopausal status and groin node positive were independent poor prognostic factors for OS.

**Conclusion:**

Groin node-positive was the only one independent poor prognostic factor for both PFS and OS. In addition, the tumor diameter longer than 25 mm. was independent poor prognostic factors for PFS while postmenopausal status was independent poor prognostic factors for OS. Special adjuvant treatment for patients with these factors should be further investigated.

## Introduction

Vulva cancer is a rare gynecologic cancer. The latest data from Globocan 2018 showed a crude rate of 1.2 per 100,000 women-year (Bray et al., 2018). The most common histology is squamous cell carcinoma (SCCA) which is classified into two types. The first type is human papillomavirus (HPV) related. This type typically is found with a high-grade squamous cell intraepithelial lesion (HSIL) and is associated with the immunosuppressive state, smoking, and usually occurs in the younger age population. The other type is non-HPV related. The precursor lesion of this type is differentiated vulvar intraepithelial neoplasia (dVIN) usually occurring in postmenopausal women (Bornstein et al., 2016). 

The standard treatment for early-stage SCCA vulva cancer is composed of the wide local or radical vulvectomy with or without groin node dissection dependent on the depth of the primary lesion. A tumor with an invasion of more than one mm., warrants groin node dissection. Adjuvant treatment with pelvic and/or vulvar radiation is given when the groin node or surgical margins were involved (Tan et al., 2019). Despite radical treatment, the recurrence rates still occurred in a range of 16-40% (Te Grootenhuis et al., 2016; Woelber et al., 2016; Meelapkij et al., 2018; Te Grootenhuis et al., 2018). It has been shown that the patients who developed recurrence revealed poor survival outcomes (Meelapkij et al., 2018; Te Grootenhuis et al., 2018). We recently reported a series of all stage of 145 SCCA vulva cancer patients who treated at our institute and found the common recurrent sites were groin and vulva regions with unfavorable survival outcomes (Meelapkij et al., 2018). Previous publications identified various clinicopathologic factors such as tumor size, depth of invasion, margin status, tumor grade, lymph vascular space invasion (LVSI), groin node involvement, and age affect the recurrence and survival outcomes (Deka et al., 2014; Woelber et al., 2016; Te Grootenhuis et al., 2018; Woelber et al., 2019). However, with small numbers in previous studies and limited data in the Southeast - Asian population and our previous study lacked of data of these independent factors. Therefore, we conducted this retrospective study to analyze the clinicopathological factors influencing the survival outcomes and included only patients who underwent surgery. This knowledge should improve our treatment guidelines for vulvar cancer patients. 

## Materrials and Methods

*Study Population*

After the protocol was approved by the local ethics committee, the medical records of the vulva cancer patients with SCCA treated at Chiang Mai University Hospital from January 2006 through December 2017 treated according to general practice guideline of our institute were reviewed. The patients who underwent vulvectomy and/or groin node dissection with at least one postoperative follow-up were included in the study. The adjuvant treatment either concurrent chemo-radiation or radiation alone was given when the groin node was positive. The patients whose vulvar specimen revealed a tumor-free margin less than 8 mm. were given radiation at the vulva site. Neoadjuvant chemotherapy was given in some patients whose primary lesions were too large. After treatment, the patients were scheduled for follow-up with history taking and pelvic examination by gynecologic oncologists every three months in the first year, every four months in the second year, every six months in the third to fifth years, and annually following. A CT-scan was performed when clinically indicated. The patients who received other treatment without surgery or never follow up after operation were excluded. 

*Outcome Measurements*

The FIGO 2009 staging, the time of recurrence or death, the clinicopathological data including menstruation status, treatment type, postoperative wound complication, underlying disease, pre-operative tumor area, groin node involvement, number of nodes removed in each side, pathologic tumor longest diameter, tumor grade, surgical margin, LVSI, and the presence of identifiable vulvar intraepithelial neoplasia or high-grade squamous intraepithelial lesion (VIN or HSIL) were searched from the medical records and pathology reports for further analysis. The detail of groin node involvement including the extracapsular node invasion was collected and were compared for survival outcome. Regarding the pathological information, all surgical pathology specimens were examined and reported by a group of gynecologic pathologists. In cases where the data was incomplete, a gynecologic pathologist (S.K.) reexamined the available histologic slides. Progression-free survival (PFS) was defined as the time from the initial treatment to the time of recurrence or progression of the disease or the time of last contact. Overall survival (OS) was defined as the same starting time as PFS to the time of patient death or the time that the patients were still alive at the end of the study. This time was sought from the Thai Civil Registration. 

*Sample Size Calculation*

The sample size was calculated by using the following formula (Lemeshow et al., 1990)

$n=\frac{Z_{1-\frac{\alpha^{p(1-p)}}{2}}^{2}}{d^{2}}$

where: 

α=Level of significance=0.05

$Z_{1-\frac{\alpha }{2}}=1.96$

*p*=Five-year overall survival of patients with squamous cell carcinoma=0.508 [6]

*d*=Level of precision=0.1 

Then a minimum sample size of 97 patients was needed.

*Statistical Analysis*

Statistical analysis of the data was carried out using IBM SPSS Statistics for Windows program (version 22; IBM Corporation, Armonk, NY, USA). A receiver operating characteristic curve (ROC) was used to assess the discriminative value and the best cut-off value of the possible clinicopathologic factors was determined to predict the recurrence or progression. The PFS and OS were estimated by the Kaplan-Meier method. Clinicopathological factors influencing survival as mentioned above were analyzed using log-rank test analysis. Cox proportional hazard models were applied to explore predictors of survival outcomes through univariate and further multivariate analysis with a P-value from the log-rank test less than 0.05. Mann-Whitney U test was used to compare the non-parametric data. A p-value of < 0.05 was considered statistically significant.

**Table 1:** Comparison of the PFS and OS Divided by Prognostic Factors

Factors	Total	5-Year PFS (%)	Univariate Analysis		Cox-Regression Analysis†			Univariate Analysis		Cox-Regression Analysis†	
			HR†	P-Value*	HR!	P-Value	5-year OS (%)	HR†	P-Value	HR!	P-Value
Menstruation status			2.58 (1.143-5.826)	0.018	2.78 (0.953-8.110)	0.061		2.47 (1.249-4.888)	0.007	2.73 (1.143-6.519)	0.024
Pre-menstruation	36	70.9					76.9				
Post-menstruation	89	44.5					52.5				
Treatment			1.588 (0.705-3.574)	0.26	-	-		1.223 (0.646-2.317)	0.536	-	-
Surgery	25	60.5					70.3				
Surgery+adjuvant treatment$	100	49					56.8				
Wound complication			1.317 (0.709-2.448)	0.382	-	-		1.002 (0569-1.764)	0.996	-	-
Not Present	87	56.9					57.2				
Present	38	48.5					64.8				
Underlying disease			1.121 (0.618-2.031)	0.707	-	-		1.513 (0.899-2.548)	0.116	-	-
Not present	60	55					66.4				
Present	65	48.7					53.5				
Tumor area (mm2)			2.678 (1.413-5.076)	0.002	1.3 (0.575-2.937)	0.529		2.344 (1.362-4.034)	0.002	1.558 (0.977-3.536)	0.059
<=11	58	67.6					75.7				
>11	67	34.8					44.4				
Groin node positive			2.79 (1.494-5.212)	0.001	2.56 (1.229-5.333)	0.012		2.526 (1.481-4.308)	<0.001	2.366 (1.283-4.363)	0.006
Not present	66	65					72.8				
Present	44	27.2					35.1				
No data	15										
Number of nodes removal (right)			0.565 (0.201-1.590)	0.273	-	-		0.605 (0258-1.415)	0.241	-	-
>=5	93	51.9					58.3				
<5	17	50.3					57.7				
No data	15										
Number of nodes removal (left)			1.115 (0.574-2.166)	0.951				0.992 (0.531-1.852)	0.98		
>=5	81	51.4					51.6				
<5	28	54.1					62.6				
No data	16										
Pathologic longest tumor diameter (mm)			3.565 (1.649-7.704)	0.001	2.639 (1.154-6.038)	0.022		2.142 (1.152-3.980)	0.014	1.152 (0.537-2.470)	0.717
<=25	47	72.5					77.8				
>25	58	40.4					48.5				
No data	20										
Factors	Total	5-Year PFS (%)	Univariate Analysis		Cox-Regression Analysis†			Univariate Analysis		Cox-Regression Analysis†	
			HR†	P-Value*	HR!	P-Value	5-year OS (%)	HR†	P-Value	HR!	P-Value
Tumor grade			1.301 (0.680-2.490)	0.425	-	-		0.694 (0.367-1.311)	0.258	-	-
Grade 1	90	60.4					57.4				
Grades 2 and 3	32	39.9					61.2				
No data	3										
Surgical margin			1.065 (0.553-2.050)	0.851	-	-		1.008 (0.569-1.787)	0.979	-	-
Negative	64	58.7					69.2				
Positive	49	37.6					58.3				
No data	12										
Nearest margin (mm)			2.637 (0.895-7.771)	0.079	-	-		1.375 (0.622-3.036)	0.432	-	-
>=3	21	83.6					81				
< 3	43	54.7					62.9				
Total	64										
VIN related			1.83 (0.921-3.635)	0.08	-	-		2.27 (1.223-4.214)	0.008	1.984 (0.908-4.334)	0.086
Present	45	51.5					74.8				
Not Present	79	47.7					51.6				
No data	1										
LVSI			1.639 (0.873-3.079)	0.12	-	-		1.389 (0.795-2.428)	0.246	-	-
Not present	87	57.3					61.8				
Present	34	33					53.7				
No data	4										

*, log-rank tes; †, Cox Proportional Hazard model: Backward Stepwise (Likelihood Ratio); !, adjusted Hazard ratio; $, adjuvant treatment: surgery followed by radiation (75 cases), surgery followed by concurrent chemoradiation (18 cases), surgery followed by chemotherapy (1), concurrent chemoradiation followed by surgery (1 case), neoadjuvant followed by surgery (4 cases); PFS, progression-free survival; OS, overall survival; HR, hazard ratio; BMI, body mass index; VIN, vulva epithelial neoplasia; LVSI, lymphovascular space invasion

**Figure 1:**
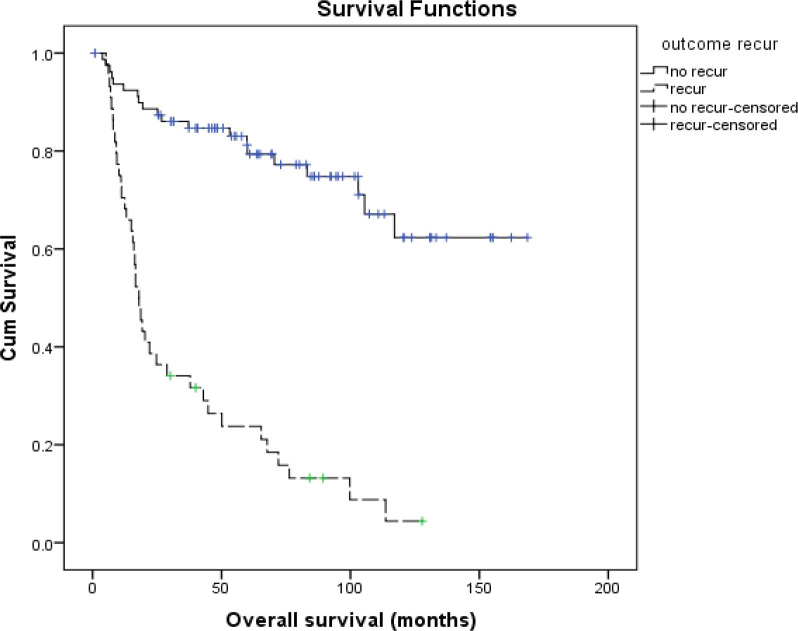
Overall Survival Divided by Recurrence Status. 5-year overall survival in no recurrence group, 79.4%; 5-year overall survival in recurrence group, 23.7%; P value < 0.001; Median FU time, 48.40 months (1-169 months)

**Figure 2:**
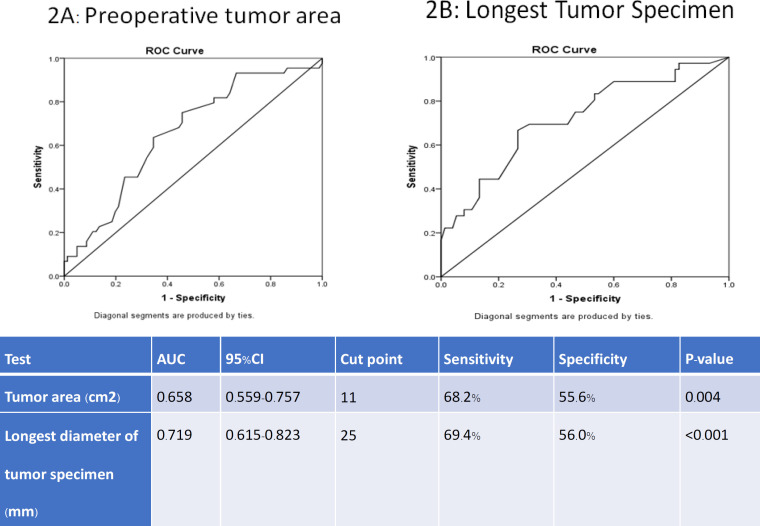
Receiver-Operating-Characteristic (ROC) and Area Under the Curve (AUC) for Clinicopathologic Parameters to Recurrence

## Results

One hundred seventy-three SCCA vulva patients were treated at our institute in the study period. Of those patients, 48 cases were excluded from the study due to non-surgical treatment in 36 cases and not followed up postoperatively in 12 cases. Therefore, 125 cases were recruited in this study. The median age of them was 57 years with a range of 32-82 years. The distribution of FIGO staging was as follow; stage I was 48 cases (38.4%), stage II was 31 cases (24.8%), stage III was 37 cases (29.6%) and stage IV was 9 (7.2%). With the median follow-up time was 15.03 months (range 1-128 months), 44 patients developed recurrence. Thus, the recurrence rate was 35.2%. At the time of analysis, 59 patients (47.2%) had died and the five-year PFS was 70%. The five-year OS rate was significantly different in patients with and without recurrence as showed in Figure 1. The patients without recurrence revealed a better 5-year OS rate of 79.4% while the patients with recurrence had a five-year OS rate of only 23.7%, P < 0.001.

Figure 2 displayed the ROC curve of preoperative tumor area, and the longest pathological tumor diameter to find the optimal cut-off point value for predicting the recurrence state. The results revealed the cut-off point level of tumor preoperative tumor area was 11 cm^2^ and the longest pathological tumor diameter was 25 mm. 

The clinicopathological data were divided into two groups for each item and compared to find the influence of PFS and OS outcomes as noted in Table 1. For PFS, postmenopausal status, pre-operative tumor area larger than 11 cm^2^, groin node-positive, and pathological longest tumor diameter more than 25 mm. were significant for poorer PFS in univariate analysis. However, only the groin node-positive and pathological longest tumor diameter more than 25 mm. were significant in multivariate analysis with a hazard ratio of 2.560 and 2.639, respectively.

Regarding overall survival, the factors including postmenopausal status, preoperative tumor area larger than 11 cm^2^, groin node-positive, pathological longest tumor diameter longer than 25 mm. and tumor without VIN were significant poor prognostic factors in univariate analysis. However, only postmenopausal status and groin node positive were significant poor prognostic factors in multivariate analysis. 

Regarding 44 patients with metastatic groin nodes, 15 cases (34.1%) revealed extracapsular invasion. The five-year PFS of patients with extracapsular node invasion was significantly poorer than patients without extracapsular node invasion (9.6% vs 38.2%, P-0.014). Also, the five-year OS of patients with and without extracapsular node invasion was 21.4% and 42.2%, respectively. However, this survival did not reach statistical difference (P = 0.265).

## Discussion

The present study found the recurrence rate as 35.2% in patients with SCCA of the vulva who were treated with surgery and those recurrent patients showed a very poor survival outcome with the five-year OS only 23.7%. This recurrence rate corresponded to previous studies. Singareddy et al., (2019) revealed 76 patients diagnosed with SCCA vulva cancer. Of those patients, 59 cases were treated with radical surgery with or without radiation while the rest were treated with radiation alone. They found a recurrence rate of 24.5% in the surgery group, 12% in surgery plus radiation, and 47% in the radiation group. Another small series from India recruited 18 SCCA of the vulva, treated with radical surgery with a recurrence rate of 27.7% (Deka et al., 2014).

Regarding the prognostic factors for survival outcome, the present study found positive groin nodes and tumor with a pathological diameter longer than 25 mm. were independent poor prognostic factors for PFS while the postmenopausal status and groin node positive were independent poor prognostic factors for OS. These prognostic factors agreed with previous studies. Li et al., (2009) revealed 184 SCCA of the vulva in Chinese patients treated with radical surgery. They reported that tumor diameter longer than two cm., lymph node metastasis, number of positive nodes, extra- nodal growth, and bilateral positive nodes were significant prognostic factors for PFS, cancer-specific survival, and OS while an age older than 60 years was a significant prognostic factor for OS. However, the authors did not report the data of multivariate analysis for these factors. Woelber et al. (Woelber et al., 2019) performed a subset analysis of AGO (Arbeitsgemeinschaft Gynäkologische Onkologie)-CaRE (Chemo and Radiotherapy in Epithelial Vulvar Cancer)-1 study (Mahner et al., 2015). The AGO-care study is a large retrospective study from 29 gynecologic cancer centers in Germany aimed to evaluate the benefit of adjuvant therapy in lymph node-positive vulvar cancer. This subset analysis recruited 1,249 patients who received groin node dissection. Of those patients, 360 (28.8%) patients developed disease recurrence within the median follow-up time of 27.5 months. The authors found the independent clinicopathological factors for vulvar recurrence were nodal involvement, presence of a residual tumor, older age, and advanced tumor stage. However, this study did not show the prognostic factors for PFS or OS. Woelber et al., (2012) reviewed 157 primary SCCA of vulva patients treated with primary surgery and found the positive lymph nodes and increasing age per year were independent prognostic factors for PFS. This data corresponded to our results with the factor of positive nodes. We did not find postmenopausal status that reflected advanced age to be an independent prognostic factor for PFS. The different outcomes might be from the non-similar recruited patients. Woelber et al., (2012) recruited only patients who received radical local resection with a surgical margin of 10 mm. and BGND while our study recruited all patients who underwent all operations. However, our study found the postmenopausal status was the independent prognostic factors for OS. This might be explained by the OS data were obtained from patients who died from the Thai Civil Registration whereas the PFS data was obtained from medical records that probably missed tumor progression data if the patients did not come back for follow-up. 

Regarding the characteristic of groin node involvement, the present study found poor survival outcomes in both PFS and OS in patients with extracapsular node involvement. This result corresponded to previous studies (Li et al.,2009; Bornstein et al.,2016). However, with the low number of patients who had positive groin nodes in our study, the difference in OS did not reach statistical significance despite the five-year OS of patients with extracapsular node involvement were very poor at 21.4% compared to those patients with intracapsular node involvement that revealed a five-year OS of 42.2%.

Regarding tumor size, a pathological tumor diameter longer than 25 mm was the independent poor prognostic factor for PFS not for OS in our study. This might be from the effect in multivariate is not strong enough for tumor diameter. Aragona et al., (2014) Study reviewed 194 patients with SCCA of the vulva and reported that the tumor size of more than 8 cm was the independent prognostic poor prognostic factor for OS. This size was larger than our result. The different values might be from the non-similar inclusion criteria. Aragona et al., (2014) Study included only patients with a pathological tumor-free margin of at least 8 mm while our study included all patients who underwent surgery. 

The OS in the patients with identifiable VIN was better than those without, but the difference was not significant in multivariate analysis. VIN is related to HPV-associated vulvar cancer, which showed a better survival outcome and more commonly occurred in premenopausal women compared to non-HPV-associated vulvar cancer (Bornstein et al., 2016; Watkins, 2019). Unfortunately, P16 immunohistochemistry to confirm the HPV-related vulvar cancer was not performed in this study. However, our study found that post-menopausal status was an independent poor prognostic factor for OS. This is probably from the non-HPV vulvar cancer type that often develops in postmenopausal women (Bornstein et al., 2016) . 

Regarding the pathological tumor-free margin, the present study did not find the pathological tumor-free margin less than 3 mm. as an independent prognostic factor for survival outcomes. Our results correspond to a study by Grootenhuis et al., (2019) that reviewed 287 SCCA vulvar patients treated in two Dutch centers and reported the ten-year local recurrence rate as 42.5%. They summarized that a pathological tumor-free margin distance did not affect the risk of local recurrence either using a cutoff of eight, five, or three mm. 

Due to various results of these prognostic factors, Te Grootenhuis et al., (2018) recently published a systematic review regarding the prognostic factors for local recurrence of SCCA of the vulva from 22 studies. They summarized these prognostic factors as follows: pathologically tumor-free margin distance less than 8 mm., presence of lichen sclerosis, groin node metastases, tumor grade, tumor size, depth of tumor invasion, LVSI, tumor localization, and presence of HPV remain equivocal due to the inconsistent outcome of these publications. 

The strength of our study was that all data came from one institute with many patients. Thus, the variety of treatment patterns was similar and all pathology was reported by gynecologic pathologists. However, with the nature of a retrospective study and some patients did not continue regular follow-up, the recurrence data might be missed. Also, we could not identify cancer-specific survival. Possibly some patients died from other causes. 

In conclusion, groin node-positive and tumor diameter longer than 25 mm. were independent poor prognostic factors for PFS while postmenopausal status and groin node-positive were independent prognostic factors for OS. Patients with these factors should be given adequate treatment and careful follow-up. 

## Author Contribution Statement

Prapaporn Suprasert Project development, Data analysis, Manuscript writing. Monwanee Muangchang Data collection Manuscript writing. Surapan Khunamornpong Data analysis, Manuscript writing. 

## Availability of Data and Materials

The datasets generated and/or analyzed during the current study are not publicly available due to patient privacy but are available from the corresponding author on reasonable request.
